# Interleukin-15 facilitates muscle regeneration through modulation of fibro/adipogenic progenitors

**DOI:** 10.1186/s12964-018-0251-0

**Published:** 2018-07-20

**Authors:** Xia Kang, Ming-yu Yang, You-xing Shi, Mei-ming Xie, Min Zhu, Xiao-long Zheng, Chen-ke Zhang, Zi-lu Ge, Xu-ting Bian, Jing-tong Lv, Yun-jiao Wang, Bing-hua Zhou, Kang-lai Tang

**Affiliations:** Department of Orthopedic Surgery, Southwest Hospital, Third Military Medical University, Gaotanyan Str. 30, Chongqing city, 400038 People’s Republic of China

**Keywords:** IL-15, Fibro/adipogenic progenitor, Fatty infiltration, Fibrosis, Muscle injury, Rotator cuff tear

## Abstract

**Background:**

Chronic muscle injury is characteristics of fatty infiltration and fibrosis. Recently, fibro/adipogenic progenitors (FAPs) were found to be indispensable for muscular regeneration while were also responsible for fibrosis and fatty infiltration in muscle injury. Many myokines have been proven to regulate the adipose or cell proliferation. Because the fate of FAPs is largely dependent on microenvironment and the regulation of myokines on FAPs is still unclear. We screened the potential myokines and found Interleukin-15 (IL-15) may regulate the fatty infiltration in muscle injury. In this study, we investigated how IL-15 regulated FAPs in muscle injury and the effect on muscle regeneration.

**Methods:**

Cell proliferation assay, western blots, qRT-PCR, immunohistochemistry, flow cytometric analysis were performed to investigate the effect of IL-15 on proliferation and adipogensis of FAPs. Acute muscle injury was induced by injection of glycerol or cardiotoxin to analyze how IL-15 effected on FAPs in vivo and its function on fatty infiltration or muscle regeneration.

**Results:**

We identified that the expression of IL-15 in injured muscle was negatively associated with fatty infiltration. IL-15 can stimulate the proliferation of FAPs and prevent the adipogenesis of FAPs in vitro and in vivo. The growth of FAPs caused by IL-15 was mediated through JAK-STAT pathway. In addition, desert hedgehog pathway may participate in IL-15 inhibiting adipogenesis of FAPs. Our study showed IL-15 can cause the fibrosis after muscle damage and promote the myofiber regeneration. Finally, the expression of IL-15 was positively associated with severity of fibrosis and number of FAPs in patients with chronic rotator cuff tear.

**Conclusions:**

These findings supported the potential role of IL-15 as a modulator on fate of FAPs in injured muscle and as a novel therapy for chronic muscle injury.

**Electronic supplementary material:**

The online version of this article (10.1186/s12964-018-0251-0) contains supplementary material, which is available to authorized users.

## Background

Muscle degeneration is widely presented in many musculoskeletal diseases, including chronic rotator cuff tear, aging related sarcopenia and Duchenne muscular dystrophy (DMD). Fibrosis and fatty infiltration are two most important features in this pathological process. The deposition of adipocytes in muscle is irreversible, which can deteriorate muscle quality and thus cause dysfunction with inferior clinical outcomes [1–4]. Interestingly, unlike fatty degeneration, fibrosis was considered to be a sign in both degenerative and reparative processes [5–7]. The mechanism regulating fibrosis and fatty infiltration in muscle degeneration is still not fully understood. In previous studies, a subgroup of mesenchymal stem cells which specifically expressing PDGFRα, named fibro/adipogenic progenitors (FAP), was identified to be bipotential to differentiate into myofibroblast or adipocyte and thus to be responsible for the fibrosis and fatty degeneration after acute and chronic muscle injury [8–10]. On the other hand, FAPs also demonstrate “a double-edged sword” effect after muscle damage. Though it causes fatty infiltration and fibrosis in injured muscle, it is critical to activate the differentiation of muscle progenitors (so called satellite cells) and promote the regeneration of muscle [8, 11].

Previous studies showed the biological behavior of FAPs was largely dependent on microenvironment. Uezumi et al. reciprocally transplanted GFP-labelled FAPs between degenerative and regenerative environment, transplanted FAPs exhibited new characteristics adapted to new environment [8]. In addition, several cytokines which secreted by immune cells after muscle damage, such as IL-4, TGF-β, TNF-α, were essential to activate the FAPs and regulate its fate [9, 12, 13]. Besides that, FAPs can also secrete IL-33 to accumulate the regulatory T cells to facilitate the repair of muscle in aging mice [14]. The dynamic crosstalk between FAPs and surrounding environment was indispensable for maintaining homeostasis in muscular pathological conditions.

Currently, skeletal muscle was regarded as an endocrine organ [15]. It can secrete a series of cytokines, termed myokines during muscle contraction. These myokines can effect on muscle fibers, surrounding tissue and cells or distal organs by autocrine, paracrine or endocrine [15–17]. Furthermore, they have a potent effect on metabolic homestasis, especially for adipose [18–20]. Several important myokines, including MSTN, IL-15, can affect the adipogenesis or reduce the fat mass [21–29]. However, it is rarely reported whether the myokines can influence the fate of FAPs after muscle damage.

Here we screened local expression of several myokines in injured muscle tissue. The level of IL-15 was identified to downregulate significantly at the occurrence of fatty infiltration. Interestingly, we also found IL-15 can stimulate the proliferation of FAPs and facilitate the regeneration of myofibers. Our results provide a new sight into the regulation of FAPs by microenvironment after muscle injury and they point to a potential underlying cause for IL-15-mediated inhibition of adipogenesis and muscle regeneration. In addition, a potential new therapy could be further considered.

## Methods

### Human subjects

Rotator cuff samples were obtained from 8 patients diagnosed with supraspinatus muscle tears. They were hospitalized to Department of Orthopedic surgery, Southwest Hospital of Third Military Medical University. All of subjects were performed surgery of arthroscopic repair in the Department of Surgery, Southwest Hospital. The baseline characteristics were listed in Supplementary Information, Additional file 1: Table S1. Samples of supraspinatus muscle were obtained. Deltoid muscle sample (about 2mm^3^) were collected as control. All of procedures were approved by the Ethical committee at Third Military Medical University, all necessary consent was obtained from all participants.

### Animal experiments

All animal experiments and procedures were approved by the Institutional Animal Care and Use Committee at Third Military Medical University. All the mice were housed in a pathogen-free environment. C57BL/6 mice at 6 to 8 weeks old were used for experiments. To induce the acute damage or fatty degeneration model in skeletal muscle, 100 μl Cardiotoxin (CTX, 10 μM in PBS, Cat. No. 160601, Zhongxin Dongtai Nano Gene Biotechnology) or 50% glycerol (*v*/*v*) was injected into tibias anterior muscle, respectively, as described previously [8]. In some experiments, recombination mouse IL-15 protein (Cat. No. 566304, Biolegend) was administrated via intramuscular (0.5 μg per TA, i.m.) or combined with SAR-20347 (25 mg/kg, i.m., Cat. No. T4210, Targetmol) injection at specific time-points.

### Cell isolation and FACS

The isolation procedure of FAPs followed the method reported in previous study [8]. Briefly, muscle from both hind limbs of 6 to 8 weeks old C57BL/6 mice were collected. The non-muscle tissue was carefully removed. Trimmed muscle were minced and digested with 0.2% type II collagenase (Cat. No. LS004176, Worthington) for 1 h at 37 °C. Muscle slurries were filtered through 100 μm and 40 μm cell strainers (Cat. No. 431752 and 431,750, BD Bioscience) in sequence. After erythrocytes were eliminated, cells were resuspended in washing buffer consisted of PBS containing 2% FBS. Then the cells were stained with antibodies for 30 min at 4 °C in the dark. The gating strategy is CD31^−^CD45^−^Integrinα7^−^Sca-1^+^PDGFRα^+^. The information of antibodies used for FACS were listed in Additional file 1: Table S2. Stained cells were analysed by FACSAria III (BD Biosciences, NJ, USA). The data was analysed by using FlowJo v10 (Flowjo, LLC., OR, USA).

### Cell culture

Freshly sorted FAPs were cultured Matrigel-coated tissue culture plates in growth medium (GM) consisting of Dulbecco’s modified eagle medium (DMEM) supplemented with 20% FBS, 1% penicillin-streptomycin and 2.5 ng ml^− 1^ bFGF (Cat. No. PHG0021, Invitrogen). After 3 days, medium was changed with fresh medium. To induce adipogenic differentiation, cells were exposed to adipogenic induction medium consisting of DMEM with 20% FBS, 0.5 mM IBMX (Cat. No. I7018-250MG, Sigma-Aldrich), 0.25 μM dexamethasone (Cat. No. D4902-25MG, Sigma-Aldrich) and 10 μg ml^− 1^ insulin (Cat. No. PB180432, Procell) for up to 3 days and adipogenic maintenance medium consisted of DMEM with 10% FBS and 10 μg/ml insulin.

### Histochemistry, cytochemistry and imaging

Fresh frozen muscle tissues were sectioned (8 μm) using a cryostat and then fixed with 4% PFA for 5 min. Tissue sections were permeabilized in 0.5% Triton X-100 (Cat. No. T8200, Solarbio) in PBS for 5 min and then blocked for 1 h at 37 °C in PBS containing 10% normal donkey serum, 3% bovine serum albumin (BSA) and 0.1% Triton X-100, and incubated with primary antibodies at 4 °C overnight, then were staining by secondary antibodies conjugated to Cy3, Alexa 555 or 488 or phalloidine conjugated to TRITC. Specimens were counterstained with Hoechst 33,342(Cat. No. C1026, Beyotime). The primary and secondary antibodies used were listed in Supplementary Information, Additional file 1: Table S2. For Oil Red O staining, cells were fixed in 4% PFA for 10 min, rinsed in water followed by 60% isopropanol. Then stained in Oil Red O in 60% isopropanol for 15 min, then rinsed in water. Cells were visualized using a fluorescence microscope IX81 (Olympus, Tokyo, Japan) equipped with a CCD camera. Confocal images of muscle sections were captured using the confocal laser scanning microscope system TCS SP8 (Leica Microsystems, Wetzlar, Germany).

### CCK-8 assay

For CCK-8 (cell counting kit 8) test, 1 × 10^4^ FAPs were stimulated by recombinant mouse IL-15 (100 ng/ml) or combined with SAR-20347 (10 μM) for 48 h in 96-well plates. Then 10 μL CCK-8 solution (Cat. No. CK04, Dojindo) per well were added into each well. 100 μL complete medium and 10 μL CCK-8 solution per well was considered as blank controls. The cells were incubated for 4 h at 37 °C, 5% CO2 in dark, then the values of OD 450 nm were measured using a microplate reader (Synergy H1, BioTek, VT, USA).

### BrdU staining

The isolated FAPs plated on slides were incubated with BrdU (10 μM, Cat. No. B8010, Solabio) at 37 °C for 2 h. Then the samples were fixed with 4% paraformaldehyde. After washed with PBS for 3 times, slides were treated with 2 N HCl at 37 °C for 30 min, then blocked with 1% BSA in PBS at 37 °C for 1 h. Then the samples were incubated with anti-BrdU antibody at 4 °C overnight, then incubated with Cy3-conjugated secondary antibody (Additional file 1: Table S2).

### Apoptosis assay

Cells were stained with FITC-Annexin V/PI (Cat. No. 401002, Bestbio) according to the manufacturer’s instructions. Briefly, cells were resuspended in 400 μL 1 x binding buffer, 5 μl FITC-Annexin V were added to the sample and incubated at 4 °C for 15 min in the dark, then 10ul PI were added to the sample and incubated at 4 °C for 5 min in the dark. The stained samples were detected by flow cytometry (BD FACSCalibur, BD Biosciences, NJ, USA).

### qRT-PCR analysis

Total RNA isolation was performed using TRIzol reagent (Cat. No. 15596026, Invitrogen) and reverse transcribed into cDNA using the RevertAid First Strand cDNA Synthesis kit (Cat. No. K1622, Thermo Fisher Scientific) according to the manufacturer’s protocol. qRT-PCR was carried out using a ABI 7500 Real-Time PCR system (Applied Biosystems, CA, USA). The mRNA expression levels were normalized to β-actin. Reactions were performed in duplicate using a SYBR kit (Cat. No. RR4420L, Takara) under following cycling conditions: denaturation at 95 °C, followed by 40 cycles of denaturation at 95 °C for 15 s, then annealing at 60 °C for 1 min. Relative target gene expression was calculated using the 2-ΔΔCq method. Specific primer sequences used for PCR are listed in Supplementary Information, Additional file 1: Table S3.

### Western blots

Cell extracts (50 μg of protein) was loaded onto a 7.5% SDS–PAGE gel and blotted on polyvinylidene fluori (PVDF) membranes (Cat. No. 1620177, Bio–Rad Laboratories). Once transblotted, membranes were blocked with 5% BSA diluted in PBS. Membranes were then incubated overnight at 4 °C in PBS containing 5% BSA with primary antibody to examine the protein expression in the lysates. The blots were then incubated with secondary antibodies labeled with HRP. Signal was detected using a scanner (ChemiDoc Touch Imaging System, Bio-Rad Laboratories, CA, USA). The primary and secondary antibodies used were listed in Supplementary Information, Additional file 1: Table S2.

### Statistical analysis

Results are showed as means±SE. Student’s *t*-test was used in comparison of two groups. For more than two groups, one way analysis of variance (ANOVA) was used. Pearson product moment correlation analysis was performed to find the correlation between IL-15 and number of FAPs or area of collagen deposition. Statistical significance was considered at *P* < 0.05. For each parameter of all data presented, *p < 0.05, **p < 0.01, ***p < 0.001, values not sharing a common small letter differ significantly (*p* < 0.05). All experiments were repeated at least 3 times.

## Results

### Expression of IL-15 downregulates significantly at the occurrence of fatty infiltration after muscle damage

**Fig. 1:**
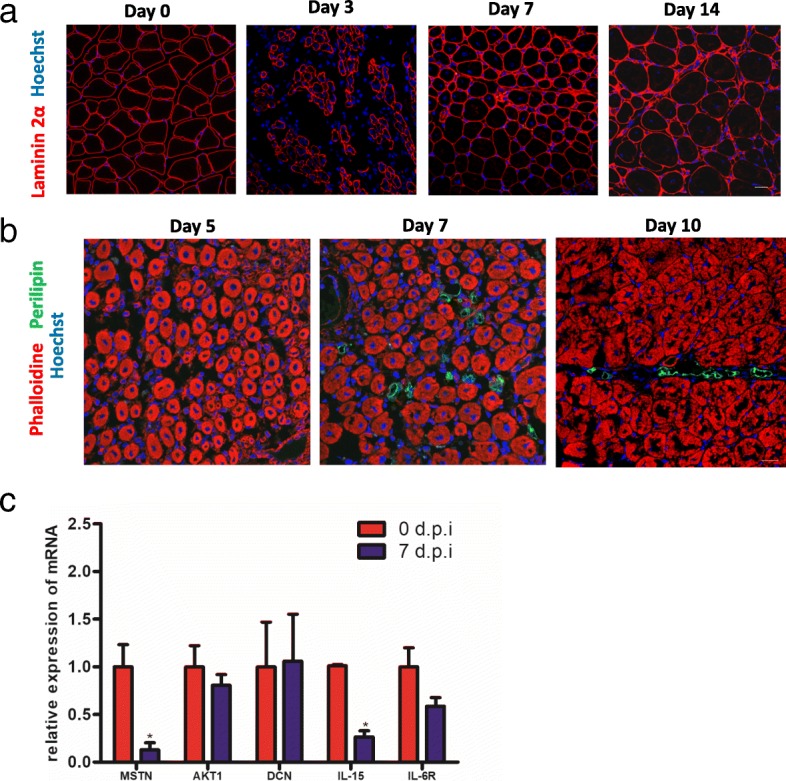
The level of IL-15 is negatively associated with fatty infiltration in chronic muscle injury. After injection of glycerol in tibialis anterior, the pathological changes of myofibers (**a**) and fatty infiltration (**b**) were examined by immunofluorescene, an antibody against adipocyte biomarker perilipin were used to detect the adipose formation in injured mice. **c** The mRNA level of candidate myokines in injured muscle on 0 days and 7 dpi. Were analyzed using real-time PCR. Scale bars, 20 μm (**a**, **b**)

Since it was reported that adipogenesis was inhibited in cardiotoxin (CTX) induced muscle injury [30], glycerol was injected into tibialis anterior (TA) muscle to induce fatty infiltration in mice. We first examined muscular pathological changes after injection of glycerol. On day 3 post glycerol injection, severe damage of myofibers can be observed and followed by myofiber regeneration on 7 dpi and 14 dpi (Fig. 1a), which was consistent with the findings in previous studies [8]. Next, we sought to confirm the time of adipose occurrence, obvious fatty infiltration can be detected on 7 dpi (Fig. 1b).To screen the myokines that can potentially regulate the adipogenesis, the mRNA quantitative analysis of common myokines at day 0 and day 7 were performed. The expression of MSTN and IL-15 were found to downregulate significantly on 7 dpi compared with on day 0 (Fig. 1c). These results suggested these two candidates may be effective on fatty degeneration in muscle injury. Since myostatin was reported to promote the FAP-derived collagen depots and inhibit the development of adipocytes [31], we focused on the effect of IL-15 on FAPs in muscle injury.

### IL-15 can prevent the FAPs differentiating into adipocytes both in vitro and in vivo

**Fig. 2:**
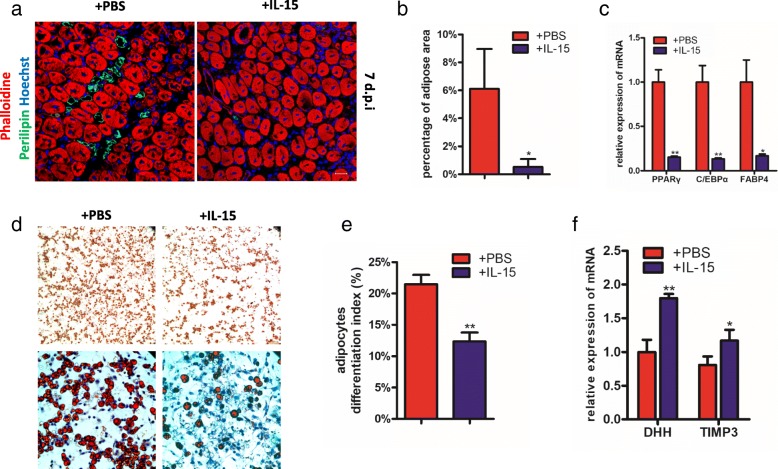
IL-15 inhibits the adipogenesis of FAPs both in vivo and in vitro. **a** Immunofluorescence for fatty infiltration after administration of IL-15 in injured muscles 7 dpi. Scale bars, 20 μm. **b** Quantifications of adipose occupied area (shown in percentage) in injured muscles 7 dpi with glycerol injection. **c** Real-time PCR for adipogenic biomarkers (c/ebpα, pparγ and FABP4) in whole injured muscles 7 dpi with glycerol injection. **d** The effect of IL-15 on adipogenesis of FAPs was detected by Oil Red O staining in vitro. Scale bars, 100 μm (top), 40 μm (bottom). **e** Quantifications of Oil Red O occupied area (shown in percentage). **f** qPCR analysis of DHH and TIMP3 in purified FAPs from injured muscles 7 dpi

Due to the mRNA expression of IL-15 was negatively associated with the occurrence of fatty infiltration (Fig. 1b and c), we investigated whether overexpression of IL-15 can prevent the adipocyte accumulation after muscle injury. The recombinant mouse IL-15 protein was injected into TA of wild-type mice that had been injected with glycerol. At day 7, the formation of adipose was largely prevented (Fig. 2a and b). In addition, level of mRNAs encoding C/EBPα, PPARγ and FABP4, the classic adipogenic markers, was significantly lower in samples administrated with IL-15 on 7 dpi (Fig. 2c). The results suggested overexpression of IL-15 can prevent the fatty infiltration in muscle injected with glycerol in vivo.

Given that FAPs were the main source of adipocytes in chronic muscle injury, we next investigated the direct effect of IL-15 on adipogenic differentiation of FAPs in vitro. Freshly FAPs were isolated using FACS following strategy as shown in Additional file 2: Figure S1. As expected, IL-15 can also prevent the adipogenesis of FAPs in vitro (Fig. 2d and e). This result suggested us that IL-15 can directly inhibit the adipogenic differentiation of FAPs and thus prevent the fatty infiltration.

Since recent study demonstrated desert Hedgehog (DHH) signaling can repressed FAP-derived adipocyte differentiation through Timp3 [30], we tested the mRNA expression of DHH and Timp3 in injured muscle with injection of IL-15. As expected, mRNA levels of DHH and Timp3 were both upregulated (Fig. 2f).

In summary, our data show that IL-15 can prevent the adipogenesis of FAPs both in vitro and in vivo, and this process is associated with the overexpression of DHH signaling.

### Overexpression of IL-15 stimulates the proliferation of FAPs through activation of Jak-STAT pathway

**Fig. 3:**
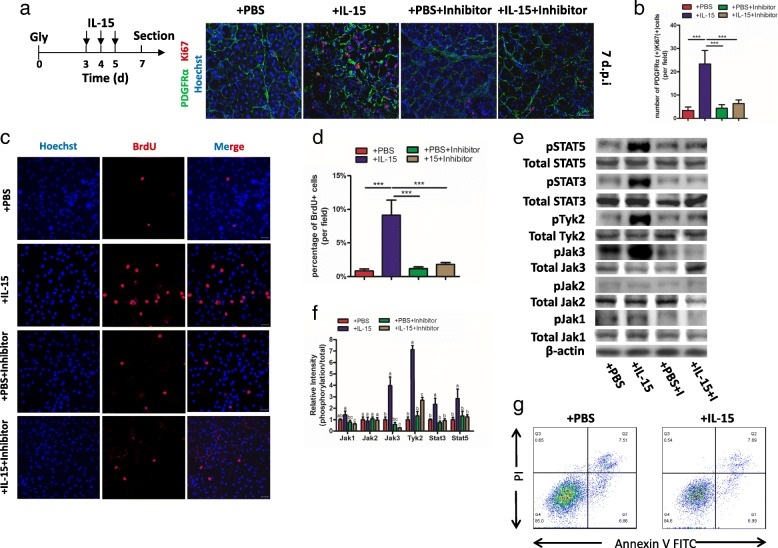
IL-15 stimulates proliferation of FAPs both in vivo and in vitro. **a** A schematic showing the experiment in vivo: IL-15 (or combination with Jak inhibitor) was administered from 3dpi to 5 dpi and samples were sectioned on 7 dpi (top). Activated FAPs were detected by staining Ki67 (bottom). Scale bars, 20 μm. **b** Quantifications of percentage of PDGFRα^+^Ki67^+^ FAPs in total FAPs. **c** The ability of proliferation of FAPs in the presence of IL-15 (or combination with Jak inhibitor) detected by BrdU staining. **d** Quantifications of percentage of BrdU^+^ FAPs in total FAPs. **e** Western blots for activation of Jak-STAT pathways after stimulated by IL-15 with/without inhibitor and (**f**) quantity analysis. **g** FITC-Annexin-V/PI assay for apoptosis of FAPs after stimulated by IL-15 for 48 h. Values not sharing a common small letter differ significantly (*p* < 0.05). Abbreviations: Gly, glycerol; I, Inhibitor

Considering IL-15 can stimulate proliferation on some cell types [32, 33], we investigated whether there was similar effective on FAPs. Since the number of FAPs decreased after 3 dpi (Additional file 2: Figure S2a and b), we preferred to injecting IL-15 for continuous 3 days from 3 dpi to observe the proliferation of FAPs clearly. Both the number of FAPs and the percentage of PDGFRα^+^ Ki67^+^ cells was largely increased in samples with administration of IL-15 (Fig. 3a and b), suggesting proliferation of FAPs was stimulated after injection with IL-15. To investigate whether it was a direct or indirect effect, we freshly isolated FAPs from muscles and stimulated with IL-15 in vitro. After cultured for 48 h, BrdU incorporation in FAPs was increased for nearly 5 folds (Fig. 3c and d). Furthermore, CCK-8 assay also revealed that treatment with IL-15 can successfully promote the growth of FAPs in comparison to controls and baseline (Additional file 2: Figure S2c).

Since Jak-STAT pathway primarily activated in IL-15-mediated cell proliferation [34], we tested whether it is also participates in IL-15-mediated proliferation of FAPs. Interestingly, we found the expression of phospho-Jak3 and phospho-Tyk2, as well as their downstream, phospho-STAT3 and phospho-STAT5, was significantly upregulated (Fig. 3e and f). In order to confirm Jak-STAT pathway regulated the proliferation of FAPs after stimulated by IL-15, we used SAR-20347, a Jak inhibitor, which can inhibit the whole members of Jak family, to block the Jak-STAT pathway. Remarkably, the proliferation of FAPs was inhibited both in vivo and in vitro (Fig. 3a-d). In summary, our results indicate IL-15 can stimulate the proliferation of FAPs through Jak-STAT pathway.

Next, we investigated whether IL-15 has effective on the apoptosis of FAPs. Samples with stimulated by recombinant IL-15 protein and controls were collected to be analyzed by apoptosis assay. No significance was observed between the two groups (Fig. 3g). The result implies the grows of FAPs stimulated by IL-15 is not due to inhibiting the process of apoptosis.

### IL-15 induces collagen deposition in injured muscle

**Fig. 4:**
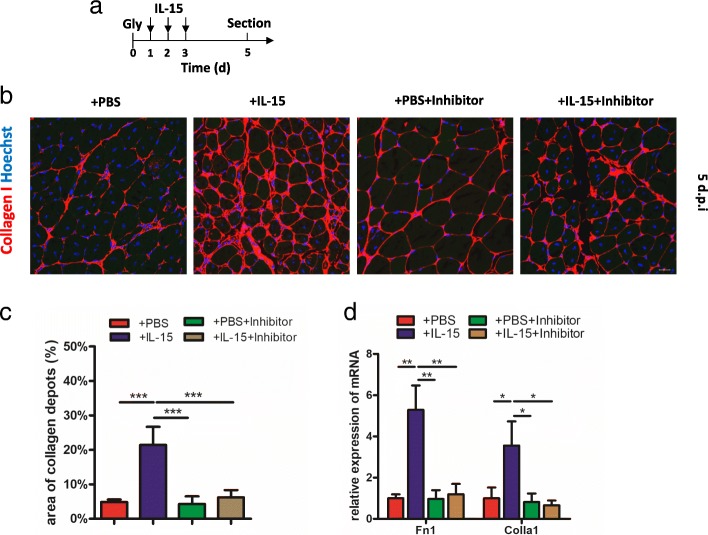
Administration of IL-15 enhances collagen deposition in injured muscles. **a** A schematic showing the experiment in vivo: IL-15 (or combination with Jak inhibitor) was administered from 1dpi to 3 dpi and samples were sectioned on 5 dpi. **b** Immunofluorescence for collagen I after administration of IL-15 in injured muscle 5 dpi. Scale bar, 20 μm. **c** Quantification of collagen deposition area (shown in percentage). **d** qPCR analysis of fibrosis-associated biomarkers, Fn1 and collagen I, in injured muscle with glycerol injection 5 dpi. Abbreviations: Gly, glycerol; Colla 1, Collagen I

In previous studies, FAPs can synthesize and secrete collagen [9, 35]. As IL-15 can stimulate the proliferation of FAPs in injured muscles, we investigated whether this promoted the fibrosis process in vivo. As expected, daily intramuscular injection of IL-15 for continuous 3 days resulted in collagen I deposition persisting in the muscle on day 5 (Fig. 4a-c). The mRNA expression of Fn1 and Collagen 1 in tissue also increased approximately 4 folds compared to controls (Fig. 4d). In addition, the fibrosis can be prevented by injection with SAR-20347 (Fig. 4b-d). The results suggest the fibrosis caused by IL-15 is associated with proliferation of FAPs.

Muscle injury with injection of CTX is a muscle regeneration model, which presents fibrosis but not fatty infiltration [9, 30], thus we also investigated whether IL-15 was effective on fibrosis in CTX injection model. IL-15 was injected at specific time and samples were sectioned at day 10 (Additional file 2: Figure S3a). Nearly 2-folds change of collagen I deposition can be found after injection with IL-15 compared with control (Additional file 2: Figure S3b and c).

Taken together, our data show IL-15 can enhance the collagen deposition in vivo after muscle damage and this process can be prevented by blocking Jak-STAT pathway.

### IL-15 facilitates muscle regeneration after injury

**Fig. 5:**
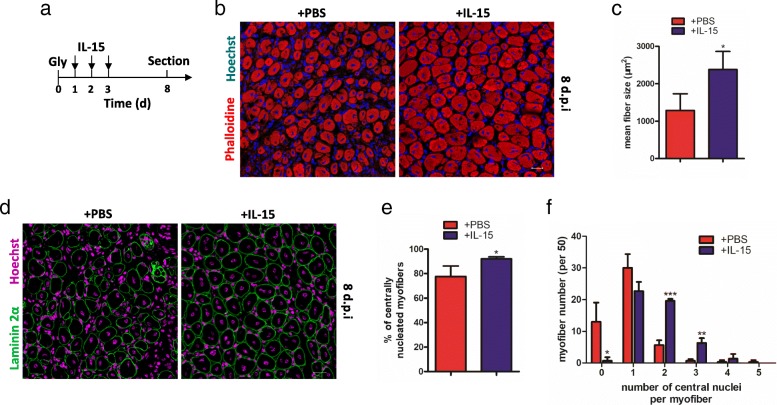
IL-15 can facilitate regeneration of myofibers in injured muscles. **a** A shcematic showing the experiment in vivo: IL-15 was administered from 1dpi to 3 dpi and samples were sectioned on 8 dpi. **b** Immunofluorescence for myofibers 8 days after glycerol injection with administration of IL-15 by staining phalloidine. **c** Quantifications of average cross-sectional area of myofibers with administration of IL-15. **d** Immunofluorescence for Laminin α2 and Hoechst in injured muscle 8 dpi with administration of IL-15. **e** Quantifications of percentages of number of myofibers with centrally located nuclei. **f** Number of nuclei present in myofibers with administration of IL-15. Abbreviations: Gly, glycerol; Colla 1, Collagen I

To investigate whether muscle regeneration was affected, recombinant IL-15 protein was injected into glycerol-injured muscle for consecutively 3 days. Then the samples on 8 dpi were collected to detect (Fig. 5a). The myofibers after injection with IL-15 was significantly larger than those injection with PBS (Fig. 5b). Quantification analysis showed cross sectional area of myofibers injection with IL-15 was nearly 2-folds larger than control (Fig. 5c). In addition, the number of centrally located nuclei is another indicator for myofiber repair. In our research, the proportion of myofibers with centrally located nuclei was significantly increased in muscle injection with IL-15 (Fig. 5d and e). On the other hand, the number of myofiber with two or three central nuclei was also more in sample injection with IL-15 compared with control (Fig. 5f). Our results showed after injecting IL-15, the regeneration of myofibers were stimulated.

### The expression of IL-15 correlates positively with numbers of FAPs and collagen deposition in patients with chronic rotator cuff tear

**Fig. 6:**
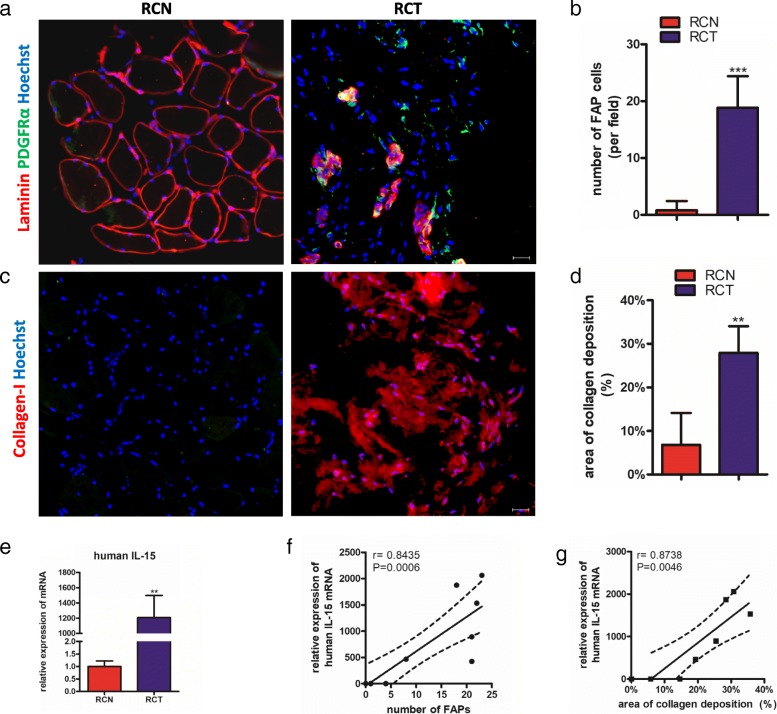
The expression of IL-15 is positively correlated with number of FAPs and collagen deposition in subjects with rotator cuff tear. **a** Immunofluorescence for PDGFRα and Laminin in muscles from subjects with RCT. **b** Quantification of number of FAPs in muscles from subjects with RCT. **c** Immunofluorescence for Collagen Iin muscles from subjects with RCT. **d** Quantification of percentage of collagen deposition area in muscles from subjects with RCT. **e** qPCT analysis of mRNA expression of IL-15 in samples from patients with RCT. **f** Pearson’s correlation analysis for mRNA level of IL-15 and number of FAPs in samples from patients with RCT. **g** Pearson’s correlation analysis for mRNA level of IL-15 and percentage of area of collagen deposition in samples from patients with RCT

To further examine the correlation between IL-15 and number of FAPs in human, we tested tissues collected from lesions during arthroscopy. Myofibers presented obvious atrophy and the number of FAPs in lesion were much more than in normal tissue (Fig. 6a and b).

In addition, the area of deposition of collagen Ishowed nearly 5-folders larger than in normal tissue (Fig. 6c and d). Next, we tested the mRNA expression of IL-15. As expected, the mRNA level of IL-15 upregulated significantly in lesion (Fig. 6e).Finally, we confirmed the expression of IL-15 in rotator cuff was positively correlated with the number FAPs and deposition of collagen I by Pearson correlation test (Fig. 6f and g).

## Discussion

Our results reveal IL-15 can inhibit the fatty infiltration and promote the muscle regeneration by regulating FAPs. IL-15 can be synthesized by myofibers in skeletal muscle, which is one of critical myokines [36]. It has been shown to reduce the white adipose tissue (WAT) mass in muscle [37], which can increase insulin sensitivity and promote lipid oxidation [38, 39]. Treatment of IL-15 can reduce the lipid deposition in human adipocytes [27]. Interestingly, IL-15 was found to regulate the differentiation of 3 T3-L1 cells, a preadipose cell line [40]. In addition, it also can inhibit the adipogenesis of stromal vascular fractions (SVFs) in vitro [29]. However, the detailed mechanism remains to be elucidated. FAPs were recently identified in muscle [8, 10]. As the primary source of fatty infiltration in injured muscle, the fate of FAPs is largely dependent on context [8, 10]. In our study, we show recombinant IL-15 protein can directly prevent the adipogenic differentiation of FAPs in vitro and thus inhibit the fatty degeneration after muscle damage. This unveils a new sight about how to prevent differentiation of preadipocytes and fatty infiltration in injured muscle. In addition, in one previous study, Hh signaling pathway can prevent the adipogenic differentiation of FAPs [30], we analyzed the level changes of Dhh pathway after administration of IL-15 and found Dhh and Timp3 were upregulated. To our best knowledge, our work firstly showed local treatment of IL-15 can enhance the expression of DHH and Timp3 in muscle and we deduces this pathway participates in the regulation of IL-15 on differentiation of FAPs.

We showed that treatment of IL-15 in injured muscle can promote the regeneration of myofibers. Myofibers were differentiated from its progenitors, so-called satellite cells (SCs), however, this process can be remarkedly regulated by cross-talking with local environment. Previous studies showed increased number of FAPs facilitate the myogenic differentiation of SCs [8] and the muscle regeneration can be impaired after blockage FAPs expansion [41], indicating FAPs are indispensable for differentiation of SCs and this process may be mediated by direct contact [8, 30]. On the other hand, Treg cells can be recruited into injured muscle by FAP-released IL-33 and promote the muscle regeneration [14]. Our data shows IL-15 stimulates the proliferation of FAPs in vitro and in vivo and thus may enhance the regulation of FAPs on the differentiation of SCs. Though the expression of IL-15 in muscle was proven to be positively associated with myofibrillar protein synthesis [42], our result provides another novel way to explain the positive effect of IL-15 on muscle regeneration. Since IL-15 can promote proliferation of immune cells by members of Jak family and its downstream, including PI3K/AKT, STAT families or ERK families [34]. Here, we confirmed that IL-15 stimulated the proliferation of FAPs through activation of Jak-STAT pathway.

In our study, we found treatment of IL-15 can enhance the fibrosis in injured muscle. The role of fibrosis in muscle regeneration is still debated. Previous studies showed collagen deposition accompanied with muscle recovery, inhibition of fibrosis was associated with impaired myogenesis while excessive fibrosis can also impair the recovey [6, 41]. FAPs were identified to participate in the pathological process importantly, they can synthesize and secrete collagen [8, 9]. As a result, the proliferation of FAPs can positively stimulate the myogenesis while enhance the fibrosis, the key point is to regulate the fate of FAPs at right time to keep the balance between regeneration and fibrosis [11]. In acute muscular injury, FAPs can be induced to apoptosis immediately after stimulating myogenesis and thus prevent excessive collagen deposition [9]. In our study, we administrated IL-15 at early stage of muscle injury, tried not to effect the apoptosis of FAPs in later stage. The results showed though the fibrosis was still enhanced, it did not impair the regeneration process remarkably. Since the duration of observation was only about 2 weeks, in our study, the influence of enhanced fibrosis caused by IL-15 on muscle function is still not fully investigated, experiments with longer time can be performed to ensure the long-term outcome.

Chronic rotator cuff tear (RCT) is commonly seen in ordinary people and athletes. Even minor RCT can cause fibrosis and fatty infiltration and thus impact outcomes of treatment [43]. Our data shows the number of FAPs and area of collagen deposition increase significantly in samples with chronic RCT compared with normal muscle. In addition, the mRNA expression of IL-15 is positively correlated with these two trends. Though we cannot get a causality between them, blocking IL-15 may release the muscular degeneration in these patients. In a previous study, Tie^+^ progenitors and PDGFRα^+^ progenitors were identified to be responsible for fibrosis and fatty infiltration in mice with RCT [44]. However, because Tie can also expressed in PDGFRα^+^ progenitors, part of Tie^+^ progenitors may belong to PDGFRα^+^ progenitors [8]. We can deduce PDGFRα+ progenitors, i.e. FAPs, are the main source to cause fibrosis and fatty degeneration in patients with chronic RCT.

In our study, we firstly explain how IL-15 regulates the fate of FAPs and thus facilitate the myogenesis in chronical muscle injury, however, there are still some limits. We established the intervention model by local injection of IL-15, usage of genetics modified animal model may be preferable. In addition, immune system has largely impact on fate of FAPs, whether the immune system participates in the regulation of FAPs stimulated by IL-15 is not investigated in this study. However, these limits don’t have significant impact on the conclusion in this study.

## Conclusions

Taken together, IL-15 can inhibit the adipogesis of FAPs while promote the muscular regeneration by stimulating the proliferation of FAPs. Our study further uncovers a new evidence how FAPs were regulated by the changes of microenvironment. In addition, due to the effect on prevention of fatty infiltration and promoting the myogenesis, IL-15 can be a candidate for therapy indicated for muscle degenerative disorders.

## Additional files

Additional file 1: **Table S1.** Participants’ Baseline Characteristics. **Table S2.** Primary and secondary antibodies used. **Table S3.** Primer sequences used in qRT-PCR. (DOCX 20 kb)

Additional file 2: **Figure S1.** Flow cytometric gating strategy for isolation FAPs using FACS. **Figure S2.** The number of FAPs dropped quickly after 3 dpi, IL-15 can promote the growth of FAPs. (a) Representative immunofluorescence images of TA sections after glycerol injection. (b) Quantification of number of FAPs in TA muscles after administration of IL-15. (c) The growth of FAPs can be stimulated by IL-15 in vitro. **Figure S3.** IL-15 enhances collagen deposition in muscle with CTX injection. (a) A shcematic showing the experiment in vivo: IL-15 was administered from 1dpi to 3 dpi after CTX injection and samples were sectioned on 10 dpi. (b) Immunofluorescence for collagen I after IL-15 injection in injured muscle 5 dpi. Scale bar, 20μm. (c) Quantification of collagen deposition area (shown in percentage). (ZIP 2164 kb)

## Abbreviations

AKT1: Serine/threonine kinase 1
ANOVA: One-way analysis of variance
bFGF: Basic fibroblast growth factor
CCK-8: Cell counting kit 8
CTX: Cardiotoxin
DCN: Decorin
DHH: Desert Hedgehog
DMD: Duchenne muscular dystrophy
DMEM: Dulbecco’s modified eagle medium
FACS: Fluorescence activated cell sorter
FAPs: Fibro/adipogenic progenitors
FBS: Fetal bovine serum
Fn1: Fibronectin 1
Gly: Glycerol
GM: Growth medium
IL-15: Interleukin-15
IL-6R: Interleukin-6 receoptor
Jak: Janus Kinase
MSTN: Myostatin
PFA: Paraformaldehyde
qRT-PCR: Real time quantitative reverse transcription PCR
RCN: Normal rotator cuff
RCT: Rotator cuff tear
SCs: Satellite cells
STAT: Signal transducers and activators of transcription
SVFs: Stromal vascular fractions
TA: Tibialis anterior
WAT: White adipose tissue
