# Supplementary material for: Interleukin-15 facilitates muscle regeneration through modulation of fibro/adipogenic progenitors
Source: Cell Commun Signal. 2018 Jul 20;16:42. doi: 10.1186/s12964-018-0251-0 (PMC6053744; doi:10.1186/s12964-018-0251-0)
Supplement: Supplementary file 1 — Table S1. Participants’ Baseline Characteristics. Table S2. Primary and secondary antibodies used. Table S3. Primer sequences used in qRT-PCR. (DOCX 20 kb) [file 12964_2018_251_MOESM1_ESM.docx]

**Table S1. Participants' Baseline Characteristics.**

| **No.** | **Age (yrs)** | **Sex** | **BMI (kg/m^2^)** | **RCT** | **Serum Glucose (mmol/L)** |
| --- | --- | --- | --- | --- | --- |
| 1 | 45 | M | 22.0 | R | 5.78 |
| 2 | 44 | F | 22.0 | R | 5.94 |
| 3 | 55 | F | 25.1 | R | 5.40 |
| 4 | 43 | F | 24.7 | L | 5.08 |
| 5 | 42 | F | 20.4 | R | 4.94 |
| 6 | 47 | M | 25.2 | L | 5.47 |
| 7 | 43 | M | 22.1 | L | 5.07 |
| 8 | 47 | F | 25.2 | R | 5.00 |

**Table S2. Primary and secondary antibodies used.**

| **Antibody** | **Clone** | **Conjugates** | **Source** | **Catalog Number** |
| --- | --- | --- | --- | --- |
| rat anti-CD31 | 390 | APC | eBioscience | 17031180 |
| rat anti-CD45 | 30-F11 | APC | eBioscience | 17045182 |
| rat anti-Sca-1 | D7 | APC-Cy7 | Biolegend | 108126 |
| mouse Intergrin α7 | 334908 | APC | R&D | FAB3518A |
| Laminin 2α | 4H8-2 |  | Abcam | ab11576 |
| Perilipin | polyclone |  | Abcam | ab3526 |
| PDGFRα | polyclone | biotin | R&D | BAF1062 |
| Ki67 | polyclone |  | Abcam | ab16667 |
| Collagen Ⅰ | col-1 |  | Santa Cruz | sc-59772 |
| anti-BrdU | 1B10E12 |  | Proteintech | 66241-1-Ig |
| human Laminin | polyclone |  | Novus | NB300-144SS |
| human PDGFRα | polyclone |  | R&D | AF307NA |
| human Collagen | EPR7785 |  | Abcam | ab138492 |
| Jak1 | 6G4 |  | Cell Signaling Technology | 3344 |
| Jak2 | D2E12 |  | Cell Signaling Technology | 3230 |
| Jak3 |  |  | Affinity | BF0256 |
| Tyk2 |  |  | Affinity | DF6851 |
| Stat3 |  |  | Affinity | AF6294 |
| Stat5 | D2O6Y |  | Cell Signaling Technology | 94205 |
| Phospho-Jak1 |  |  | Affinity | AF2012 |
| Phospho-Tyk2 |  |  | Affinity | AF7288 |
| Phospho-Jak2 | D4A8 |  | Cell Signaling Technology | 8082 |
| Phospho-Jak3 | D44E3 |  | Cell Signaling Technology | 5031 |
| Phospho-Stat3 |  |  | Cell Signaling Technology | 9134 |
| Phospho-Stat5 | D47E7 |  | Cell Signaling Technology | 4322 |
| beta-Actin |  |  | Affinity | AF7018 |
| Streptavidin |  | PE | Biolegend | 405203 |
| Donkey polyclonal anti-goat IgG |  | AF488 | Jackson ImmunoResearch | 705545147 |
| Donkey polyclonal anti-rat IgG |  | Cy3,AF488 | Jackson ImmunoResearch | 712165150,  712545150 |
| Donkey polyclonal anti-rabbit IgG |  | AF488,AF555 | Abcam | ab150073,  ab150074 |
| Donkey polyclonal anti-mouse IgG |  | Cy3 | Jackson ImmunoResearch | 715165150 |
| Anti-rabbit IgG |  | HPR | Cell Signaling Technology | 7074 |
| Phalloidine |  | TRITC | Yeason | 40734ES75 |

**Table S3. Primer sequences used in qRT-PCR.**

| **gene** | **Genbank accession number** | **forward** | **reverse** |
| --- | --- | --- | --- |
| MSTN | NM_010834.3 | TCACGCTACCACGGAAACAA | AGGAGTCTTGACGGGTCTGA |
| AKT1 | NC_000078.6 | AAGCACCGTGTGACCATGAA | GCGACGATGACCTCCTTCTT |
| DCN | NC_000076.6 | GCGCTCACGCAGTGAAAC | TAACTATGCAGCCCAGGCAA |
| IL-15 | NC_000074.6 | GTGCATCTCCTTACGCGCT | GGTFFATTCTCTCTGAGCTGT |
| IL-6R | NC_000069.6 | CGGCCTTCCCTACTTCACAA | TGCCATTGCACAACTCTTTTCT |
| PPARγ | NC_000072.6 | TCTTCCATCACGGAGAGGTC | GATGCACTGCCTATGAGCAC |
| C/EBPα | NC_000073.6 | CAAGAACAGCAACGAGTACCG | GTCACTGGTCAACTCCAGCAC |
| Fabp4 | NM_024406.3 | AAGGTGAAGAGCATCATAACCCT | TCACGCCTTTCATAACACATTCC |
| Dhh | NM_007857.5 | CGATGGCTAGAGCGTTCAC | GTACCCAACTACAACCCCGA |
| TIMP3 | NM_011595.2 | TAGACCAGAGTGCCAAAGGG | CCAGGATGCCTTCTGCAAC |
| Fn1 | NM_010233.2 | GACCCTTACACGGTTTCCCA | TGGCACCATTTAGATGAATCGC |
| ColⅠ | NC_000077.6 | CGATGGATTCCCGTTCGAGT | GAGGCCTCGGTGGACATTAG |
| human IL-15 | NM_172175.2 | GGATTTACCGTGGCTTTGAGTAATGAG | GAATCAATTGCAATCAAGAAGTG |
