# Supplementary material for: Interleukin-15 facilitates muscle regeneration through modulation of fibro/adipogenic progenitors
Source: Cell Commun Signal. 2018 Jul 20;16:42. doi: 10.1186/s12964-018-0251-0 (PMC6053744; doi:10.1186/s12964-018-0251-0)
Supplement: Supplementary file 2 — Figure S1. Flow cytometric gating strategy for isolation FAPs using FACS. Figure S2. The number of FAPs dropped quickly after 3 dpi, IL-15 can promote the growth of FAPs. (a) Representative immunofluorescence images of TA sections after glycerol injection. (b) Quantification of number of FAPs in TA muscles after administration of IL-15. (c) The growth of FAPs can be stimulated by IL-15 in vitro. Figure S3. IL-15 enhances collagen deposition in muscle with CTX injection. (a) A shcematic showing the experiment in vivo: IL-15 was administered from 1dpi to 3 dpi after CTX injection and samples were sectioned on 10 dpi. (b) Immunofluorescence for collagen I after IL-15 injection in injured muscle 5 dpi. Scale bar, 20μm. (c) Quantification of collagen deposition area (shown in percentage). (ZIP 2164 kb) [file 12964_2018_251_MOESM2_ESM.zip › Supplement figure.pptx]

## Slide 1
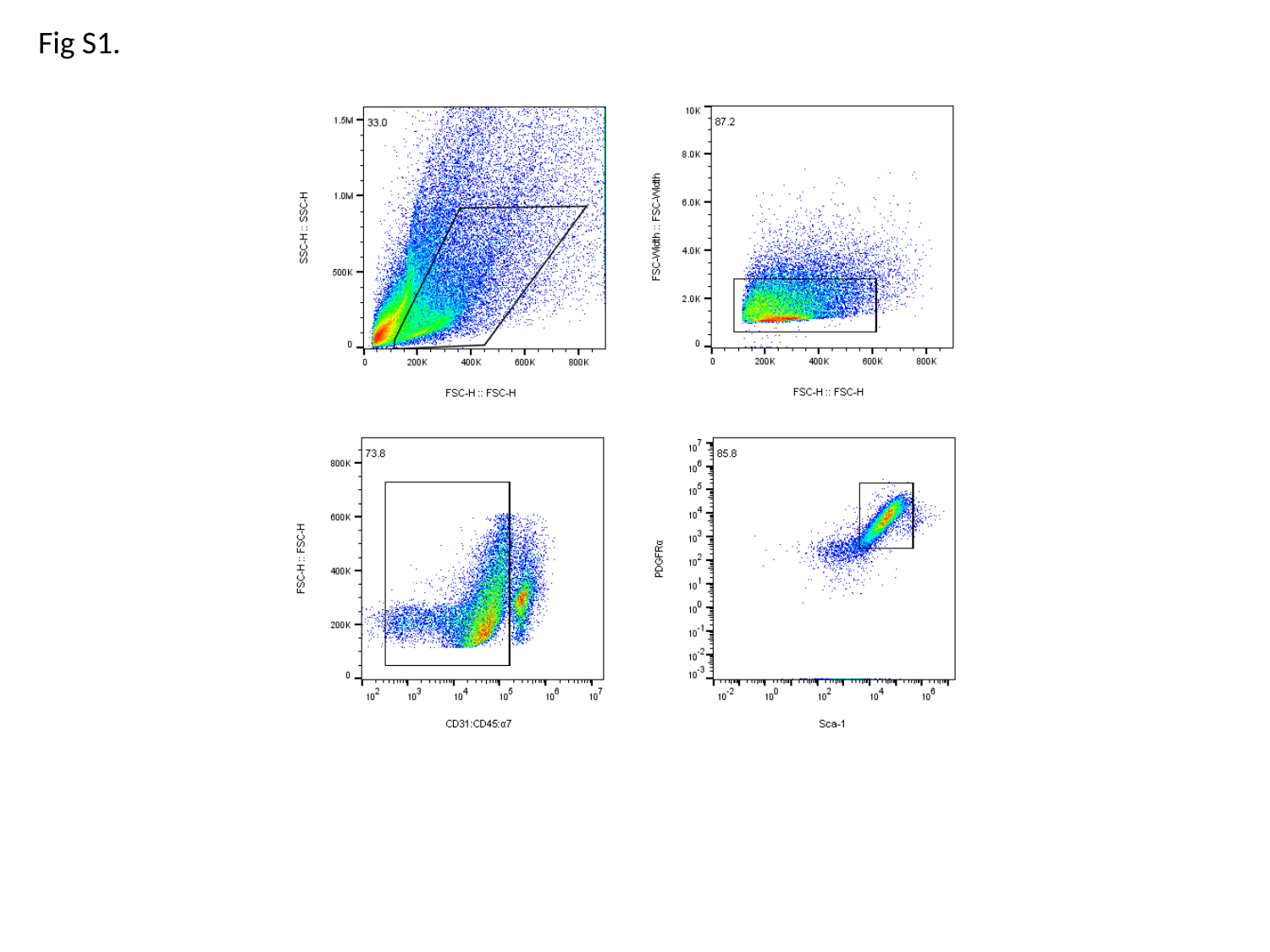

Fig S1.

## Slide 2
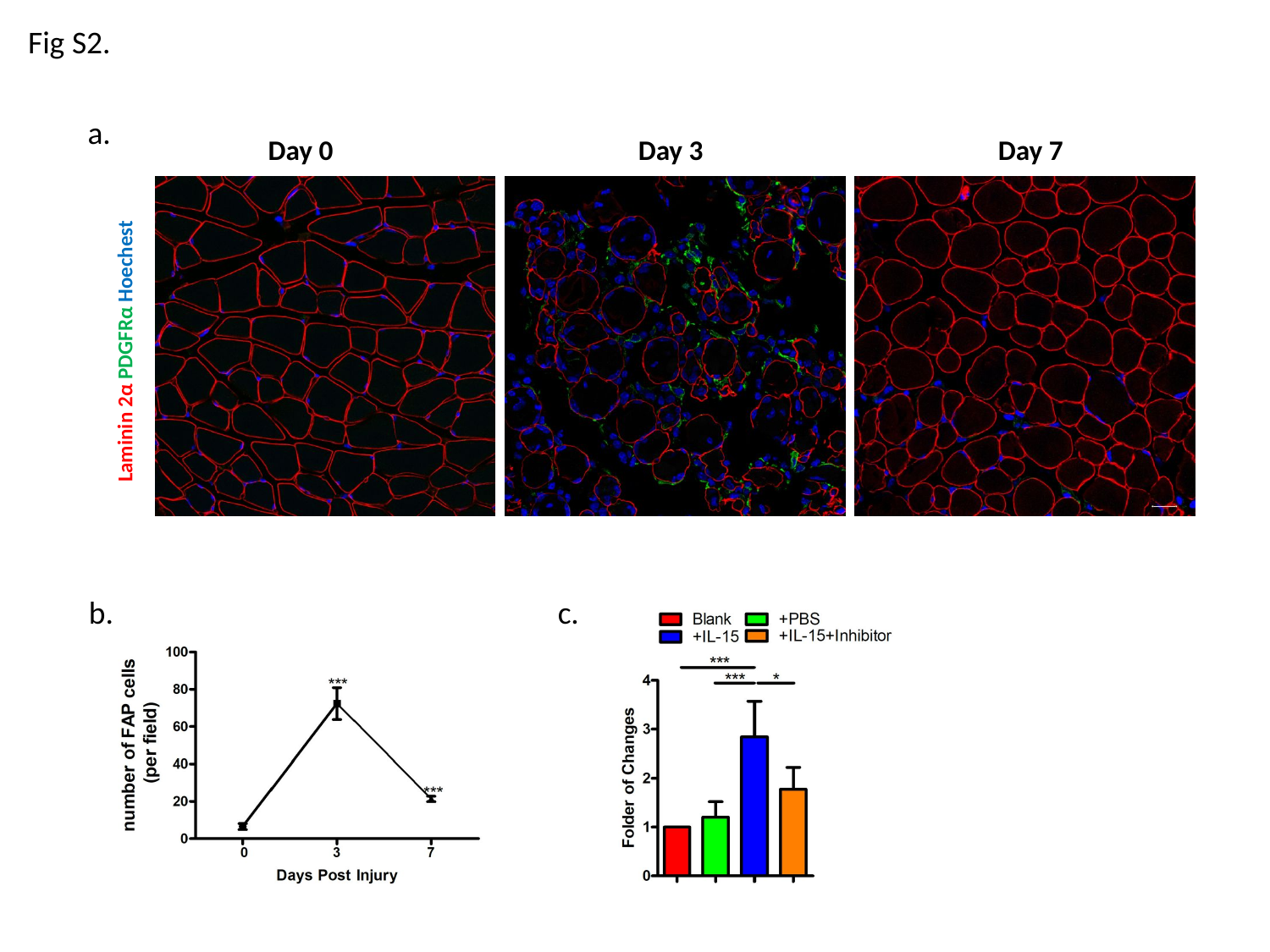

Fig S2.
a.
Day 0
Day 3
Day 7
Laminin 2α PDGFRα Hoechest
b.
c.

## Slide 3
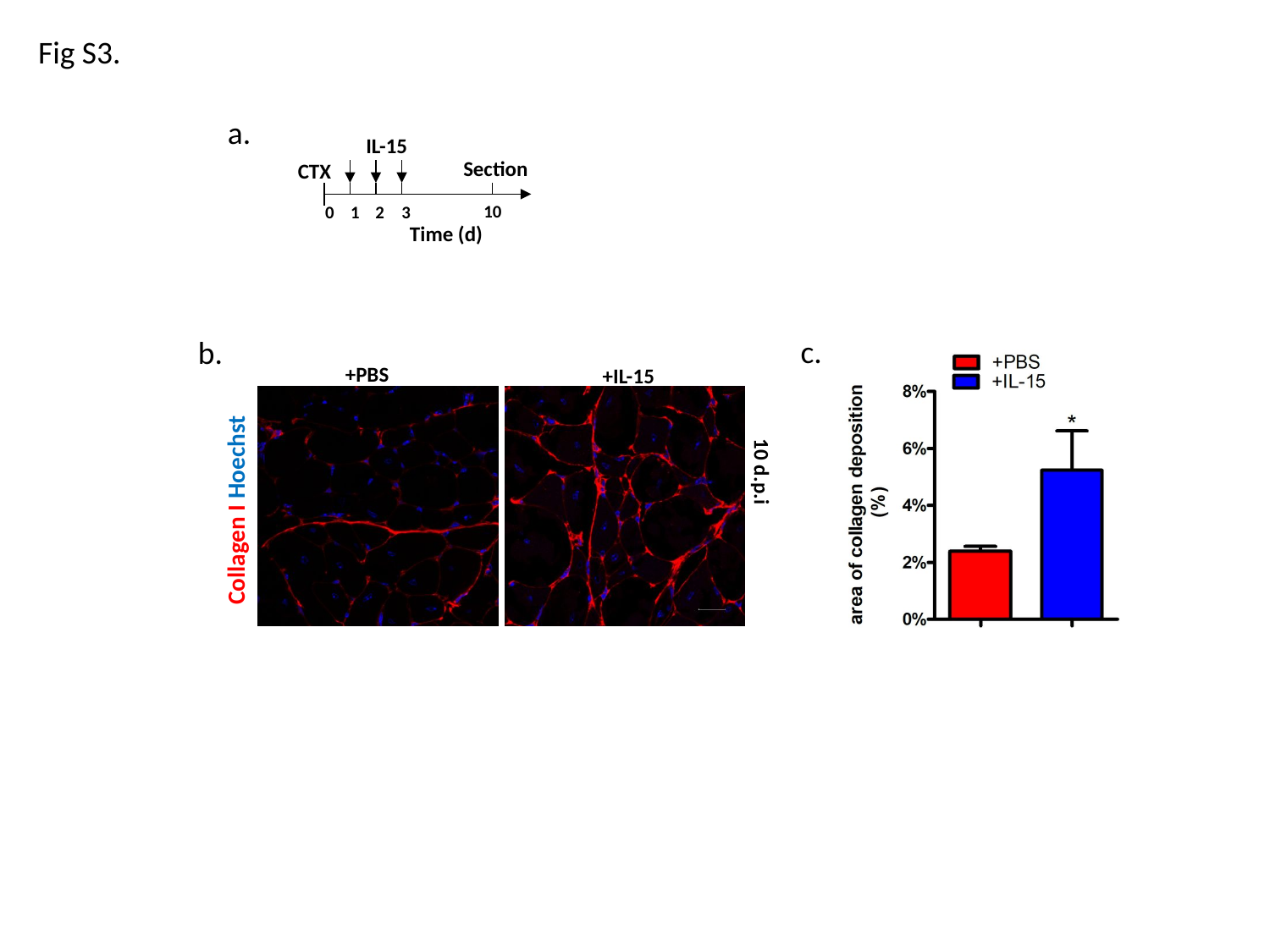

Fig S3.
a.
IL-15
Section
CTX
10
2
0
3
1
Time (d)
c.
b.
+PBS
+IL-15
10 d.p.i
Collagen I Hoechst
